# Exploring Bystander Behavior Types as a Determinant of Workplace Violence in Nursing Organizations Focusing on Nurse-To-Nurse Bullying: A Qualitative Focus Group Study

**DOI:** 10.1155/2024/4653042

**Published:** 2024-07-05

**Authors:** Kyoungja Kim, Scott Seung Woo Choi, Cheongin Im

**Affiliations:** ^1^ Department of Nursing College of Medicine Inha University, Incheon, Republic of Korea; ^2^ School of Nursing The University of Texas at Austin, Austin, TX, USA

## Abstract

**Aim:**

This study explored and analyzed the characteristics of bystander types of workplace violence in hospital nurses experiencing horizontal (nurse-to-nurse) violence. The primary research question was “What are the behavioral patterns of bystander types in peer-to-peer violence situations among hospital nurses?”.

**Background:**

Workplace violence is a result of environmental and structural conflicts, rather than deviant individual perpetrators. Research examining workplace violence suggests that bystanders are not merely witnesses of acts of aggression but can play a substantial role in escalating or deflecting violence. Types of bystander influence power dynamics within a group, resulting in changes in the pattern of violence.

**Methods:**

Employing a qualitative design, this study conducted focus group interviews with nurses from three tertiary hospitals. The qualitative data collected was analyzed using inductive and qualitative content analysis methods.

**Results:**

Nine focus group interviews (*n* = *26*) were conducted on bystanders' experiences of workplace violence. A total of 185 analysis units were identified and categorized into three main themes, based on their impact on workplace violence (reinforcing, avoiding, and suppressing) with six subcategories (facilitative reinforcer, diffuse reinforcer, condoning avoider, powerless avoider, empathic suppressor, and interventional suppressor).

**Conclusions:**

This study delineates a typology of bystander roles in workplace bullying/horizontal violence among nurses, identifying three distinct types of bystanders. The outcomes of workplace violence vary, based on the type of bystander involved as well as the dynamics among bystanders, perpetrators, and victims. *Implications for Nursing Management*. Nursing organizations should educate nurses about the concept of bystanders as this will help nurses understand that even if they may not be perpetrators or victims of workplace violence, they are still implicated as bystanders. Additionally, nursing organizations and leaders should empower nurses to play a positive bystander role.

## 1. Introduction

Workplace violence between coworkers is defined as “an act of causing another employee physical or mental harm or degrading the working environment by taking advantage of one's position or relationship in the workplace beyond the scope appropriate for work” [1]. Workplace violence typically targets a particular person, has clear intentions, is recurrent and ongoing, is likely to last for various time periods, and causes long-term job stress [2]. Globally, the prevalence of workplace violence in healthcare is as high as 87.4% [3], with 67.3% and 64.9% of health-care institutions reporting incidents of workplace violence in North America and Asia, respectively [4]. In Canada, approximately one-third of nurses with less than three years of experience are exposed to workplace violence weekly or daily [5]. The frequency of workplace violence increased during the COVID-19 pandemic [6], making it a prevalent and serious issue in hospital settings.

Workplace bullying among nurses is a critical problem and a significant risk factor that aggravates patient safety in clinical fields. According to previous studies, workplace violence can lead to more frequent medication errors, communication impairment, and lower quality of care [7–10]. More directly, workplace violence among hospital nurses impairs their physical and psychological health and leads to increased turnover [7–10]. Given the crucial negative consequences of workplace violence, global concerns about workplace violence in clinical settings are gathering attention. Studies have shown that workplace violence can be aggravated not only by the perpetrator's personal disposition and characteristics but also by work environmental factors, such as heavy nursing workload and ineffective systems [11, 12]. Therefore, some aspects of workplace violence should be considered the result of environmental and structural conflicts rather than individual perpetration. Another significant characteristic of workplace violence in nursing context is that most nurses are aware of its existence [13], making it a group issue [10, 14].

In violent situations within a social group, especially between coworkers, the majority often act as witnesses rather than being directly involved as victims or perpetrators. In such social violence scenarios, the negative consequences extend beyond just the victim and perpetrator to also affect the witnesses. In a study examining university students' responses to violent situations on campus, most witnesses felt unsafe and experienced negative consequences from the violence [15]. In nursing contexts, similar phenomena occur when individuals witness violent situations. In Báez‐León and colleagues' work [16], most nurses reported feeling uncomfortable and nervous during violent situations, even though they were neither victims nor perpetrators [16]. These negative consequences of violence have been referred to as ripple effects [17] or covictimization [18]. In one previous study, approximately 22% of nurses who witnessed workplace violence considered resigning from their nursing job [17]. Therefore, workplace violence is not solely a dyadic problem involving victim and perpetrator but also a triadic issue that includes witnesses.

Bystanders encompass more than just witnesses or observers; they are individuals who, as members of a social group, can choose their own behavior in response to violence occurring within the social group [18]. In school violence, bystanders are classified as assistants, reinforcers, outsiders, and defenders [19] and further subdivided into bully, puppet-master, victim, avoidant, abdicator, sham, and helpful bystander [20]. Paull et al. [18] classified bystanders of workplace violence into constructive and destructive types and further subdivided them into 13 types depending on whether they were active or passive: instigating, manipulating, collaborating, facilitating, abdicating, avoiding, succumbing, submitting, empathizing, intervening, defusing, sympathizing, and defending.

Bystander in social groups plays a key role because violence patterns can be influenced by the predominant bystander type [18], which in turn affects the power dynamics within the group [18, 21]. For instance, facilitators, such as assistants or reinforcers, encourage and participate in violence by the perpetrator [22], thereby reinforcing the frequency and intensity of the violence [16, 23, 24]. In contrast, victim advocacy types, such as defenders, have a positive effect by supporting the victim and inhibiting the perpetrator's violence [22]. Abdicating types, such as outsiders, ignore or avoid violent situations, resulting in increased isolation for the victim within the organization [18, 22, 25].

However, there is a lack of studies on bystanders of workplace violence in hospital nursing settings [14]. This gap may persist in part due to the long-standing social tolerance of excessive tension and interpersonal power imbalances within hospital nursing organizations. At times, these aggressive customs are deliberately utilized to immerse novice nurses in the organizational culture [26]. The tolerance for and complicity of nursing organizations in violent situations often render nurse silent witnesses to such incidents in workplace because they accepted it as a norm; nurses may perceive such behavior as the norm within the collective nurse workgroup, thereby normalizing workplace violence as part of their socialization process [27]. In the same vein, some argue that workplace violence among nurses correlates with either the victim's perceived lack of work competence [28] or the characteristics of the nursing organization [29], invoking the oppressed group theory. In this context, nurses may find it easy to witness workplace violence without intervening, as they have not been adequately supported to adopt any role other than that of an abdicator [30]. Moreover, they have never been trained to effectively intervene as appropriate bystanders and address instances of violence [31].

Thus, further research is needed to establish a foundational understanding of how bystander types within nursing organizations differ from those observed in other social groups. In cases of peer-to-peer violence in nursing workplaces, bystanders influence each other's behavior [18, 22]. Additionally, considering that witnessing violence in the workplace is common in clinical settings, more transparent and detailed qualitative data can be gathered more appropriately through group interviews than in one-on-one interviews. Empirical research suggests that focus groups may provide more appropriate settings to elicit sensitive personal information than individual interviews [32]. Thus, in this study, we employed the focus group interview method to facilitate participants with shared experiences to engage in in-depth discussions on the given topic and to share their diverse perspectives through mutual interaction [33]. Therefore, this study aimed to explore and analyze the characteristics of bystander types of workplace violence between hospital nurses in clinical settings. The research question was “What are the behavioral patterns exhibited by bystander types in instances of workplace violence between hospital nurses in clinical settings?”

## 2. Materials and Methods

### 2.1. Research Design

We adopted a qualitative study design using focus group interviews and qualitative content analysis to explore the types and characteristics of bystanders of workplace violence between hospital nurses.

### 2.2. Research Participants

The study population comprised clinical nurses working in general tertiary hospitals. Morgan [33] suggested a total of 18 research participants in three groups, with a maximum of six people per group, for general focus group interview research. However, during the data collection period in 2021, the national quarantine guidelines for COVID-19 in Korea permitted only small group gatherings of five or fewer people. Consequently, with a research team consisting of two individuals, the plan was to have three research participants per group, totaling six groups. Considering a 20% dropout rate due to the COVID-19 pandemic, the target sample size was 22 nurses.

The inclusion and exclusion criteria were developed considering the *fittingness* of qualitative research. The inclusion criteria for nurses were as follows: (1) provided direct nursing care to inpatients, (2) had more than one year of clinical experience as of the interview date, and (3) worked continuously without leave of absence for more than six months as of the interview date. The exclusion criteria were as follows: (1) had less than one year of clinical experience as of the interview date, (2) were nursing managers who did not provide direct nursing care, and (3) were outpatient and operating room nurses with different nursing delivery systems.

### 2.3. Data Collection Method and Procedure

We collected data from three university hospitals, each located in one of Korea's major cities: Seoul, Incheon, and Suwon. The selected hospitals had over 1,000 beds, a ratio of beds to nurses ranging from 2.0 to 2.5. Participants were recruited through a research recruitment notice that included the research team's information as well as the purpose, background, and methods of the study. Informed consent was obtained. When dealing with sensitive topics such as workplace violence, group participants should share similar backgrounds and experiences to create a safe and comfortable environment for them to disclose their personal experiences [34]. To construct the focus group, the research team discussed the criteria related to participants' heterogeneity and decided that these should include clinical seniority. To date, knowledge of the clinical nurses' workplace violence experience and bystander behaviors differ from unit characteristics and clinical seniority. [35, 36]. Drawing from existing literature, the research team structured each focus group to comprise members from similar departments but with diverse levels of clinical seniority. To ensure the *fittingness* of the qualitative research, participants were allocated to the focus groups based on the similarity of hospitals and departments, as well as the diversity of their clinical careers. In total, nine groups were formed, each consisting of three participants. No new information emerged after the ninth focus group interview, indicating that saturation was achieved. The data were collected between July and August 2021.

In the focus group interview, one facilitator (the first author, RN, PhD, female) and one assistant (the second author, RN, PhD, male, or the third author, RN, MSN student, female) attended the discussions with the participants. The facilitator was a nursing researcher who had 15.6 years of experience as a hospital nurse with theoretical sensitivity for understanding workplace violence and the bystander phenomena in the clinical field. The assistants were two nursing researchers with experience in qualitative research. Prior to the interviews, neither the facilitator nor the assistants had any relationship with any of the participants. The participants could get information about the research team through the recruitment notice.

The focus group interviews were conducted outside each hospital in a private, secure setting with a round table, chairs, and refreshments. Each interview was audio-recorded and lasted between 51 and 62 minutes. In total, the nine sessions amounted to 425 minutes.

The focus group research guidelines outlined by Morgan [33] were followed. First, the operational rules and guidelines for the focus group interviews were established. Initially, the facilitator introduced the research team and the reasons for their interest in this topic. Field notes were taken during the interviews by the assistants. Second, the facilitator participated in all discussions, listened to the contents, and asked the research participants additional questions when necessary. Third, to ensure *credibility*, the facilitator and the research assistants reviewed the recordings and took debriefing notes immediately after the focus group interview. If required, the research team reconfirmed the content of the statements. Repeated interviews were carried out for two participants via phone separately. Recordings were immediately transcribed, and the transcripts were validated through member checks. The transcripts were returned to two participants for comments to ensure their reliability and integrity. Fourth, after the focus group interview, the transcripts were analyzed considering words, context, frequency, intensity, and pattern of repeated statements.

### 2.4. Focus Group Interview Questions

The focus group interview questions were developed through a literature review. As outlined by Paull et al. [18], a bystander is an individual who takes action in violent situations. Furthermore, the behaviors exhibited by other bystanders can serve as triggers or inhibitors for the actions of another bystander [18, 22]. Consequently, the main research questions were structured to inquire about the diverse bystander behaviors observed by the participants, contextual situations surrounding these behaviors, and resulting outcomes and consequences.

The focus group interviews began with introductory questions about the experience as bystanders of workplace violence, after informing participants of the concept of bystanders of workplace violence. The interviews began with low-level structured questions, and then, a funnel strategy was used. The interview questions are presented in Table 1. At the end of the interview, the facilitator summarized the contents of the discussion and confirmed the meaning and intention of the statements.

### 2.5. Data Analysis

The collected data were analyzed using inductive and qualitative content analysis methods. Qualitative content analysis allowed for a systematic approach to analyzing the qualitative data collected [37]. It operated within the interpretative framework of the hermeneutic paradigm [38], acknowledging multiple subjective realities and emphasizing the mutual construction of data, which led to the development of individual and multifaceted perceptions of the phenomenon [39, 40]. Through the qualitative content analysis, complex and rich data from research phenomenon were analyzed moving, beyond descriptive categories to uncover underlying meanings inherent in these categories, thereby identifying latent and interpretative content and formulating themes and subthemes [38, 41]. The inductive content analysis method proved suitable for exploring phenomena that may lack comprehensive prior explanations or knowledge [42]. It involved deriving insights directly from the collected raw data [43], thereby enabling researchers to extract direct information without imposing preconceived theoretical perspectives [42]. In every step of data analysis, all authors (including the facilitator and interview assistant) read the transcripts repeatedly, coded the data independently, and reached a consensus through discussions.

### 2.6. Preparation Phase

The preparatory stage began by selecting units of analysis to categorize the stated content, such as words, phrases, and sentences [39]. With decontextualization and recontextualization, this nonlinear analysis provided an opportunity to organize codes based on similarities and differences in the data, thus facilitating the transition to the next stage where data are separated from the context [41]. This approach allowed for the illumination of all participants' experiences of the phenomenon [41]. During the condensing and coding phase, decontextualization was carried out [37]. Each researcher repeatedly read the transcribed data and obtained a sense of the whole content. Then, we set the criteria for selecting the analysis unit through discussion. Two experienced authors independently classified the analysis units: Researcher 1 (the first author) extracted 191 analysis units and Researcher 2 (the second author) extracted 161 analysis units. After two rounds of discussion, 185 analysis units were extracted.

### 2.7. Organization Phase

The organization step comprised open coding (e.g., indexing), categorization, and abstraction of the extracted analysis units [39]. The abstracting process encompassed all types of collected data, including attitudes and perceptions [37]. Reorganization and recontextualization were carried out during the organization phase [37, 39]. During recontextualization, separated utterances were merged into a new pattern along with their contextual information, which contributed to the abstracting [44]. During the organization phase, a hierarchical structure was established by grounding into categories and subcategories at the various levels of abstraction [38]. To conduct this process, Researchers 1 and 2 independently coded the qualitative data using the extracted 185 analysis units. Open-code data were categorized into several groups using a comparative analysis and a consensus process. The abstraction and naming of each category were discussed several times, and three categories and six subcategories were derived. As an abstraction, a description of the generated categories and naming using content characteristic words was formulated. Based on the categories established during this process, Researcher 1 outlined the types and characteristics of hospital nurses' bystander behaviors in workplace violence.

All phases of data analysis were iterative and cyclical. The data analysis process was consensus-based in which all three researchers participated. The process and data analysis results were reviewed by a nursing professor, a doctoral hospital nurse with 32 years of clinical and rich qualitative research experience, and two research participants.

### 2.8. Trustworthiness (Rigor)

Based on the criteria outlined by Lincoln and Guba [40], all interviews were recorded and transcribed; vague or inaccurate transcript contents were reconfirmed with the research participants; two study participants validated them by phone. For *fittingness*, the participants were nurses working in a general tertiary hospital with various departments, work characteristics, and careers. A sufficient number of interviews were conducted until the data reached saturation. The final results were reviewed by two participants. The data collection process was recorded and stored to ensure auditability and confirmability. In addition, the research team consisted of experts in qualitative research methodology. Two qualitative research experts (a nursing professor with qualitative research experience and a clinical nurse with 32 years of experience and a doctorate) reviewed the validity of the results. Subsequently, the results of the final analysis were summarized.

### 2.9. Ethical Considerations

This study was conducted in accordance with the ethical standards of the Helsinki Declaration after obtaining ethical approval from the Institutional Review Board of Inha University (Inha University Hospital Institutional Review Board No. 2021-03-009).

## 3. Results and Discussion

### 3.1. Study Participants

A total of 26 nurses (including two from the research team) participated in the focus group interview, excluding one who had contracted COVID-19. Participants' (*n* = 26) average age was 26.04 ± 1.31 years. The majority were women (*n* = 24, 92.3%). Their average length of clinical experience as nurses was 39.08 ± 11.03 months. All were registered nurses and staff nurses working in general units (*n* = 16), intensive care units, or emergency rooms (*n* = 10) (Table 2).

### 3.2. Contents Analysis

In the preparation stage of data analysis, the research teams transcribed and read the data repeatedly and understood the overall meaning. Based on this, the research team set the criteria for the codes of analysis according to participants' responses to workplace violence. During the analysis process, the research team agreed that the participants' bystander behavior was influenced by how they perceived workplace violence and the difference in clinical career between the perpetrator, victim, and themselves (bystanders). Additionally, bystander behavior was found to influence workplace violence in departments.

**Figure 1:**
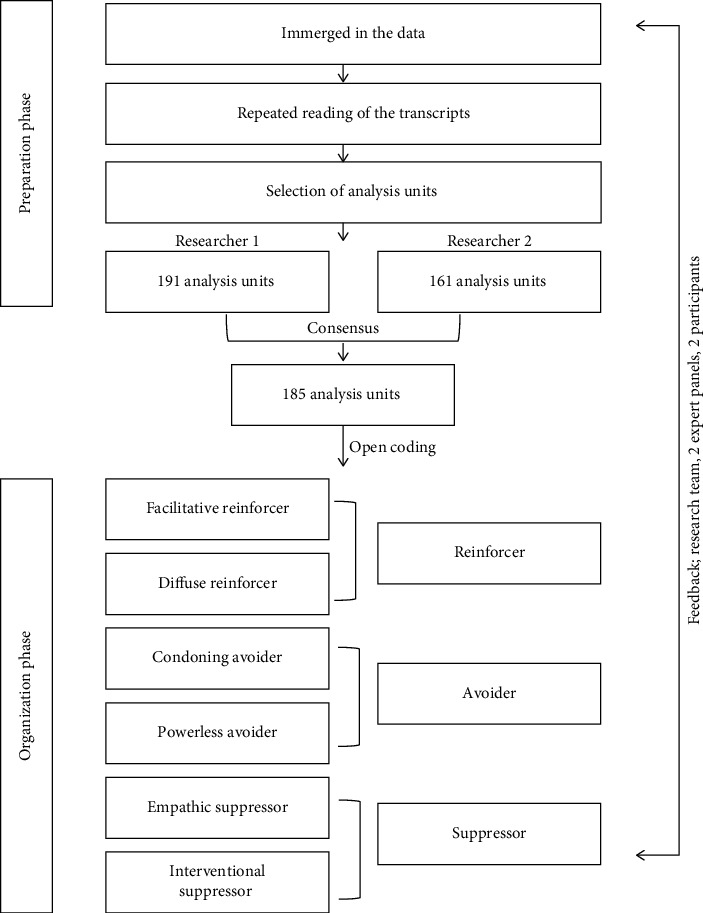
Data analysis flowchart.

Based on this, 185 analysis units were extracted according to the analysis criteria and classified into three major themes (reinforcing, avoiding, and suppressing) and six subcategories (facilitative reinforcer, diffuse reinforcer, condoning avoider, powerless avoider, empathic suppressor, and interventional suppressor) (Table 3) (Figure 1). Focus group interviews and data analysis were conducted in Korea to ensure data quality. The participant quotes illustrating the findings were translated into English by the second author, a bilingual nursing researcher with extensive clinical experience in the U.S.

### 3.3. Reinforcer

Participants indicated that bystanders could play a role in reinforcing bullying behavior in clinical settings. Two types of reinforcers were identified: facilitative and diffuse.

This type of behavior was generally observed in nurses with a higher level of experience than the victim and with a higher or equal level of experience than the perpetrator. Therefore, nurses with less experience were more likely to be negatively affected by workplace violence.

#### 3.3.1. Facilitative Reinforcer

The participants suggested that there is a type of bystander who does not instigate but joins in an act of bullying in the clinical setting. Facilitators adversely affect victims by actively involving themselves in bullying behaviors and are likely to have the same or even higher organizational authority as perpetrators. A facilitator is more likely to collaborate with or defend a bully when they are closely related to the bully.

“It really frustrates me, you know, say I am being bullied by a senior staff member, then some friends of the bully see us and walk over to us, and say things like “do not go easy on her. She has got to learn her lesson.”

(Participant 2, Group 1)

“It pisses me off when other people [the bully's friends] chime in or laugh with [the bully] when I am being bullied; it is like I am having multiple bullies at the same time.”

(Participant 1, Group 3)

“Sometimes they [bystanders] say, “Why do you make this so hard for my friend?” while laughing with the bully…Then, I now fear not just the bully but the whole group that the bully hangs out with.”

(Participant 2, Group 6).

#### 3.3.2. Diffuse Reinforcer

Participants suggested that bystanders reinforce bullying by telling other members of an organization about what happened between the perpetrator and the victim. Bystanders of this type reinforce bullying by gossiping in a way that makes the bullying incident look like something that is part of everyday clinical life. Consequently, an organizational climate may be established that perceives workplace aggression as a normative behavior.

“When a bullying incident occurs, there are always people who like spreading the news to others… especially during handoffs at a change of shift… they talk about it as though it is not that big of a deal.”

(Participant 1, Group 7)

“I really do not want to join their gossip… but it is practically hard for me to say flat out, “I do not want to engage in gossip” to a group of seniors and rain on their parade … so, I would just find an excuse to stay out of it.”

(Participant 1, Group 9).

### 3.4. Avoiders

Participants described bystanders as those who avoided taking action when a bullying event occurred. Two types of avoidant bystanders were identified: condoners and powerless.

This type of bystander behavior was described by nurses with all levels of experience. They mentioned that this type of bystander behavior is observed among nurses who have already accepted workplace violence as part of their daily lives. Acceptance is considered an inevitable form of job training, especially for nurses early in their career. This kind of behavior was also demonstrated when individuals opposed workplace violence but felt powerless to take action because they believed nothing would change.

#### 3.4.1. Condoning Avoider

Condoning avoiders seemed to believe that bullying was an inevitable part of training. They consider bullying to be some kind of a rite of passage or initiation for new nurses. They go so far as to believe acts of bullying are acceptable when they occur between junior and senior staff, assuming that juniors should learn their lessons the hard way.

“Well, I think it [bullying targeted at junior nurses] is inevitable in clinical settings… some mistreatment may be necessary on the job… if you are a new nurse and want to be a good one… you might need abusive tricks from seniors.”

(Participant 2, Group 3)

“I do not want to see that [bullying behavior by others] … I would just leave the scene [without intervening] … She [the perpetrator] might have a good reason to be hard on her [victim] … and maybe she [victim] deserves it.”

(Participant 3, Group 4).

#### 3.4.2. Powerless Avoider

Participants identified a type of bystander who did not condone bullying but refused to take action because of fear of becoming a target. They would try to leave the scene of the incident or turn a blind eye toward it. These bystanders, who were often junior nursing staff members, expressed great dissent and distress regarding the workplace violence. As illustrated in the following extracts, the power difference between the bystander and perpetrator is an important factor that renders the bystander powerless to intervene.

“She [the perpetrator] is senior to me; what do you think I can do about it [bullying]? Nothing! Absolutely nothing. I cannot really step in when it is a senior [harassing a junior]. I walked away and returned.”

(Participant 1, Group 2)

“I have never seen anyone stop [in the situation] or come forward, but what can I do when I have little experience?”

(Participant 2, Group 8)

“Sometimes I feel like I am being scolded, and I hate it so much. There is nothing I can do. This is because no one came out.”

(Participant 1, Group 8).

### 3.5. Suppressor

The suppressor category reflects bystanders who support victims. They intervene in bullying incidents. Nevertheless, they are always wary of bullying in the workplace and do whatever they can. Two subcategories are emerged as follows: empathic and interventional suppressors.

These intervention measures are typically only available to nurses with extensive experience. The recommended intervention methods include engaging in a situation between the perpetrator and the victim, stopping the situation, or redirecting the perpetrator's focus. However, in the case of nurses with less experience than the perpetrator, this behavior was described as an expression of sympathy and support for the victim rather than a direct action against the perpetrator.

#### 3.5.1. Empathic Suppressor

Participants indicated that bystanders talked to victims privately after the bullying incident and offered them emotional support. These bystanders allowed the victims to know that they were aware of the ongoing bullying incidents in the unit and that they cared about the victims' feelings. However, they did not take any action to help victims when the bullying occurred. Empathic suppressors were at lower career levels than bullies in the order of seniority based on length of employment.

“When I see one of the new nurses being reprimanded by senior staff, I try to look away. It is hard for me to intervene in such a situation… but at the end of a shift I try to find her [the victim] and cheer her up … like patting her on the back and saying things like “You are doing great! - I had to go through this too when I was a new nurse.”

(Participant 1, Group 4).

#### 3.5.2. Interventional Suppressor

Participants referred to bystanders who stepped in to stop the bullying act. These bystanders understood the destructive consequences of bullying for the organization. They seem to believe that bullying incidents in a clinical unit increase the stress level of not just the junior staff but also that of the workplace in general. Upon recognizing a bullying incident, they immediately step in and find out what happened by listening to the story from multiple sources, both perpetrators and victims, separately. Most interveners are perceived as being in a position of power in the organizational hierarchy.

“There are instances in which I happen to be the most senior nursing staff member on a shift. If I saw someone bullying another day, I tried to intervene. Previously, I did not care. Now I just tell her “Take it easy!” or call her by her full name like “Ms. so-and-so.” This usually does the trick.” (Participant 2, Group 9)

## 4. Discussion

This study explored types of bystanders of workplace violence between hospital nurses through qualitative research using focus group interviews. Hospital nurses' bystander behaviors in nurse-to-nurse workplace violence were classified into three types based on their actions: reinforcer, avoider, and suppressor.

The reinforcing bystanders who intensify workplace violence are categorized into facilitating and diffuse reinforcers. Facilitating reinforcers aggravate the negative consequences of workplace violence by sympathizing with perpetrators' violent acts and diffuse bystanders make workplace violence commonplace by spreading rumors about it throughout the organization. In this study, nurses in high-power positions within the group displayed reinforcing bystander-type behaviors. Similar to other types of social violence, workplace violence is triggered by a power imbalance among group members [26]. Power imbalance in the clinical field arises from differences in seniority, actual position, and clinical career experience [26, 45]. In these circumstances, it is easy to inflict workplace violence through job training or advising nurses who are new or in a lower career position [46]. In this study, many participants reported that they were exposed to or had observed workplace violence between nurses during job training as novices or advanced beginners. This type of job training has long been recognized as a socialization process unique to nursing organizations, in which workplace violence is recognized as a daily ritual by all perpetrators, victims, and bystanders and has become a social norm [26]. Social norms are the stated or implied rules or standards of conduct that apply to collective behavior [45]. The participants of this study described the reinforcing bystander behaviors as “that kind of thing has always happened.” Workplace violence between perpetrators and victims is “intentional and target-oriented” [26, 46]; reinforcing bystanders do not target specific people in everyday life. As a nonspecific act, their behavior can be seen as a long-adopted norm of the collective nurse workgroup rather than an act of intentional violence to deliberately suppress the victim. However, the act of strengthening the harm as a reinforcing bystander is accepted as violence by victims and other witnesses, especially by nurses in lower career positions. Hutchinson et al. [47] suggested that organizational tolerance for workplace violence is an important factor influencing bullying behaviors. By allowing the development of a pattern of repeated violent behavior, organizational tolerance and acceptance of workplace violence lead to a new form of intensified aggression. According to Paull et al. [18], aggressive bystander acts increase group tolerance for violence and, hence, reinforce the social norm of accepting workplace violence. This reinforcement worsens the power imbalance within the organization and makes witnesses more fearful of becoming the next victim, exacerbating violence in the group.

The second bystander type identified in this study is the avoider type, who may condone the violence or feel powerless and avoid the situation. Condoning bystander behaviors were observed in members of all levels of clinical careers within the group. “Doing nothing” is the most frequently reported behavior among witnesses of various social violence [14, 48]. This is also observed in nursing organizations, and inaction has been reported as the most common behavior of nurses when witnessing workplace violence [14, 16]. Báez‐León et al. [16] highlighted that, when most nurses witness workplace violence, they choose not to engage in it or to remain silent. This was expressed as ignoring, taking no action, or doing nothing [30]. The reasons for being a condoning bystander are different from those for other forms of social violence. Hoxmeier et al. [48] found that the most common reasons for avoiding violent situations among college students were “none of my business” or “unsure of the situation.” However, one reason for condoning bystander behavior in the nursing context is the perception of workplace violence as a natural phenomenon in nursing groups. In particular, the participants reported that they did not get involved “because I learned how to do the job that way,” and sometimes, they avoided violent situations “to let it happen.” In particular, this was reported prominently in workplace violence during the job training of nurses in lower career positions. Participants who experienced or chose the condoning bystander behavior said that those in a lower career position needed to learn the nursing job “with a certain level of tension.” Therefore, rather than recognizing workplace violence as a problematic situation, it was recognized as a natural or necessary situation. However, powerless bystanders of workplace violence were different from those who condoned violence. They admitted that workplace violence was prevalent but did not accept that it was natural or justified. Instead, they believed that it could not be prevented or felt helpless to stop it and avoided it. Learned helplessness contributes to neglect of workplace violence in nursing organizations [14, 16]. Another cause of powerless bystander behavior identified in this study was fear of retaliation. Fear of reprisal has been reported in previous studies as the reason for inaction against workplace violence in nursing organizations [16, 49]. The absence of reprisal fear was a critical influential factor in nurses' intention to help victims of workplace violence [16]. In a previous study, the reasons for reluctance to act against workplace violence were reported as fear of negative influence on oneself (39.7%) and cases in which the perpetrator was too powerful (35.5%) [49]. Fear of reprisal grew stronger when the perpetrator retaliated [24, 50]. Consequently, witnessing violence in the workplace acts as another form of violence for powerless bystanders, and they share the ripple effect of the harm caused by violence [51]. Avoiding bystander behaviors strengthens organizational tolerance for violence in the group by making the victim, as well as witnesses, perceive violence as a natural organizational characteristic [16, 52], resulting in the isolation of victims and the strengthening of violence in organizations [10, 18].

The third type of bystander identified in this study was the suppressor, which was divided into empathic and interventional suppressors. Nurses who understood that workplace violence was an unnecessary immoral practice and an unjust behavior that adversely affected both nurses and patients exhibited this type of behavior. They were classified as groups that expressed sympathy for the victims' situation and groups that chose actions to support them. Empathic suppressors paid attention to workplace violence but were characterized by supporting the victim after the situation was over rather than stopping the incident. As a result, the negative consequences of workplace violence experienced by victims were mitigated; however, the influence of workplace violence prevention was weak. Empathic suppressors were nurses with lower power than perpetrators. They expressed that if the perpetrator had a longer clinical career, they could not stop the perpetrator. In contrast, an interventional suppressor directly engaged in workplace violence and interrupted or stopped the situation to protect the victim and restrain the perpetrator. Interventional suppressors had a higher career status than perpetrators. Suppressor bystanders positively influence workplace violence and its consequences for nursing organizations. Positive bystander behavior had a more significant influence on nursing outcomes than workplace violence did. According to previous studies, when there was a positive perception of bystanders, nursing care quality was better [10] and there were fewer handover errors [53]. Suppressor bystanders not only mitigate the negative consequences of workplace violence by protecting the victim but they also dampen the power imbalance between the perpetrator and the victim by creating group pressure on the perpetrator to stop such incidents [10, 18]. By informing group members that workplace violence is not acceptable, suppressor bystanders can influence organizational norms that tolerate workplace violence [10, 18, 52]. Consequently, the frequency and intensity of workplace violence can be reduced and prevented [18, 22, 25]. Considering that nurses in a higher career position can form supportive subjective norms and reduce fear of reprisal, suppressor bystanders are crucial. Baez-Leon et al. [16] reported that the right action of a witness can be an important motivator for others. MacCurtain et al. [49] also emphasized the influence of positive bystanders, stating that positive bystander behaviors of nurses in higher career status with important positions in the department can refix socially constructed norms. In this regard, organizational strategies to strengthen positive bystanders can be an effective approach to prevent workplace violence [14, 54, 55].

This study has several limitations. First, it employed focus group interviews rather than in-depth individual interviews as the source of qualitative data. This was done because the research team intended to bring out the participants' various experiences regarding workplace violence, including interactions within the interview group. There might have been limitations in deriving the in-depth experience of each individual during this process. Therefore, in future research, the three types of bystander behavioral experiences presented in this study need to be explored more deeply. Second, a limitation of the current study arises due to the research context of the COVID-19 pandemic. The qualitative data were collected in 2021, during the pandemic period. The research team kept in mind that the workplace environmental changes caused by the COVID-19 pandemic could present another bias in the exploration of workplace violence phenomenon. To minimize the potential influence of COVID-19, the research team strived to keep the discussion within the general and comprehensive range of workplace violence and bystander behavior. Additionally, all interviews were conducted on topics unrelated to COVID-19, focusing on the phenomenon itself. Another major change faced by the research team due to the COVID-19 pandemic was the size of the focus group, which included only three participants, owing to the COVID-19-related restrictions. Considering this limitation, we encouraged the participants to interact with each other sufficiently during the interview and were able to reach saturation by involving a sufficient number of groups. Third, the current research participants were recruited from the big tertiary hospitals in the metropolitan cities of South Korea, so the external validity could be limited, and the results should be generalized with caution. To address this limitation and consider the fittingness of the qualitative research, the research team tried to strike a balance between participants' homogeneity and heterogeneity. For the heterogeneity of the participants, clinical seniority was considered an individual difference, and for the homogeneity, the hospital and department were considered to have contextual similarities. Fourth, this study did not utilize a theoretical framework as the basis for constructing its research methodologies. Therefore, further research should employ a useful theoretical framework to explain workplace violence among nurses and its consequences. For example, affective events theory, which posits that workplace events trigger affective reactions that in turn influence employees' attitudes and behaviors [56], could be considered. Fifth, workplace violence experienced by nurses can be categorized as patient-nurse, doctor-nurse, and nurse-nurse. This study explored bystanders' involvement in workplace violence by focusing on violence between nurses. As confirmed in this study, the type of behavior of bystanders in workplace violence is influenced by the individual's perception of the violence (accepted social norms, etc.) and power imbalance between perpetrators, victims, and bystanders. Thus, nurses' bystander behavior in patient-nurse and doctor-nurse violence may differ from the results of this study. Based on these results, future studies can develop a measurement tool that can measure bystander types in the workplace in a nursing context. A quantitative study is recommended to empirically describe the influence of a positive bystander on the perpetrator, victim, and other bystanders.

## 5. Conclusions

This study explored specific types of bystanders unique to the nursing context. Three different types of bystanders were identified and categorized by the perceptions of workplace violence as accepted natural norms and power differences in clinical careers between perpetrators, victims, and bystanders. In addition, each type of bystander had a different influence on perpetrators, victims, and other bystanders, thereby aggravating or suppressing workplace violence. This suggests that a strategy can be proposed to overcome the limitations of existing workplace violence prevention policies which focus only on violence between perpetrators and victims and to prevent workplace violence more fundamentally, targeting multiple bystanders. Therefore, nursing organizations must more actively establish strategies that utilize the concept of positive bystanders to prevent violence in the workplace. Specifically, nursing organizations should educate nurses about the concept of bystanders so that the nurses understand that, even if they are not a perpetrator or victim of workplace violence, as bystanders, they are still involved. Additionally, nursing organizations and leaders should empower nurses to play a positive bystander role.

## Figures and Tables

**Table 1:** Focus group interview questions.

Category	Questions
Introductory questions	“Have you ever experienced or witnessed violence at work?”
	“When there is violence at work, how do you and your colleagues react?”
	“What was your experience of your coworker's behavior in a violent situation at work?”

Main questions	“Please tell us your experience of various bystander behavior at workplace violence.”
	“Under what circumstances do you or your colleagues exhibit bystander behavior?”
	“How do you and your peers experience changes in behavior as a result of bystander behavior?”

Closing questions	“Do you have any more comments to share on workplace violence and bystander conduct?”
	“The following is a summary of the interviews conducted so far. Do you agree with us? Or is there anything else that requires clarification or correction?”

**Table 2:** Demographic characteristics of participants (*n* = 26).

Characteristics	Modalities	*n* (%) or Mean ± SD
Age (years)		26.04 ± 1.31
	≤25	10 (38.5)
	26∼30	16 (61.5)

Gender	Women	24 (92.3)
	Men	2 (7.7)

Marital status	Unmarried	25 (3.8)
	Married	1 (96.2)

Education	Bachelor's	24 (92.3)
	Master's and above	2 (7.7)

Hospital size (beds)	≥2,000	8 (30.7)
	1,000–2,000	6 (23.1)
	≤1,000	12 (45.2)

Length of clinical experience (months)		39.08 ± 11.03
	13–36	10 (38.5)
	37–60	16 (61.5)

Job position	Staff nurse	26 (100.0)

Unit type	General ward	16 (61.5)
	ICU/ER	10 (38.5)

ICU = intensive care unit; ER = emergency room; SD = standard deviation.

**Table 3:** Types of bystander response behavior against workplace violence.

Categories	Subcategories	Description
Reinforcer	Facilitative reinforcer	Involves themselves in the act of bullying; believes that tense and violent situations are natural and necessary in clinical setting to keep nurses alert
		A facilitative reinforcer deliberately contributes to the perpetuation or exacerbation of bullying behavior. Their behaviors exacerbate the harmful climate that the bully has cultivated, and thus, enhance workplace violence
	Diffuse reinforcer	Spreads gossip about the bullying incident; believes that tense and violent situations are natural and necessary in clinical setting to keep nurses alert
		A diffuse reinforcer supports the violent climate by bringing up the witnessed workplace violence incident to other nurses causing them to feel that bullying is normal in the clinical field. Their behaviors exacerbate the harmful climate and, thus, enhance workplace violence

Avoider	Condoning avoider	Takes no action to step into or resolve the situation; believes that tense and violent situations are natural and necessary in clinical setting to keep nurses alert
		Condoning bystanders may downplay the severity of the situation or deny that workplace violence occurs
		Their behaviors exacerbate the harmful climate and enhance workplace violence
	Powerless avoider	Takes no action toward bullying despite their belief that violent behavior is wrong and detrimental to the organization. However, powerless bystanders do not intervene in the act or support the victim because of fear of retaliation or lack of experience or empowerment. As a result, their behaviors exacerbate the harmful climate and enhance workplace violence

Suppressor	Empathic suppressor	Checks in with victims and provides emotional support after an incident; believes that violent behavior is wrong and detrimental to the organization. An empathic suppressor may listen to victims and assist them in getting the required help. Thus, they reduce the negative consequences of workplace violence
		These types of bystanders indirectly provide perpetrators with a signal that workplace violence is unacceptable, which in turn can deter workplace violence
	Interventional suppressor	Uses words or actions to directly intervene in the bullying situation or distract the bully; believes that violent behavior is wrong and detrimental to the organization
		An interventional suppressor may physically intervene to stop perpetrators, alert authorities, or assist the victim. These types of bystanders directly provide perpetrators with a signal that workplace violence is unacceptable, which in turn can deter workplace violence

## Data Availability

The interview data supporting the findings of this study are available from the corresponding author upon request, for researchers who meet the criteria for accessing confidential data.
